# Short Communication: Choosing the Right Program for the Identification of HIV-1 Transmission Networks from Nucleotide Sequences Sampled from Different Populations

**DOI:** 10.1089/aid.2020.0033

**Published:** 2020-11-06

**Authors:** Nicholas Bbosa, Deogratius Ssemwanga, Pontiano Kaleebu

**Affiliations:** ^1^ Medical Research Council/Uganda Virus Research Institute and London School of Hygiene & Tropical Medicine Uganda Research Unit, Entebbe, Uganda.; ^2^ Uganda Virus Research Institute, Entebbe, Uganda.

**Keywords:** HIV-1, HIV-TRACE, Cluster Picker, transmission network, cluster, pair

## Abstract

HIV-TRAnsmission Cluster Engine (HIV-TRACE) and Cluster Picker are some of the most widely used programs for identifying HIV-1 transmission networks from nucleotide sequences. However, choosing between these tools is subjective and often a matter of personal preference. Because these software use different algorithms to detect HIV-1 transmission networks, their optimal use is better suited with different sequence data sets and under different scenarios. The performance of these tools has previously been evaluated across a range of genetic distance thresholds without an assessment of the differences in the structure of networks identified. In this study, we tested both programs on the same HIV-1 *pol* sequence data set (*n* = 2,017) from three Ugandan populations to examine their performance across different risk groups and evaluate the structure of networks identified. HIV-TRACE that uses a single-linkage algorithm identified more nodes in the same networks that were connected by sparse links than Cluster Picker. This suggests that the choice of the program used for identifying networks should depend on the study aims, the characteristics of the population being investigated, dynamics of the epidemic, sampling design, and the nature of research questions being addressed for optimum results. HIV-TRACE could be more applicable with larger data sets where the aim is to identify larger clusters that represent distinct transmission chains and in more diverse populations where infection has occurred over a period of time. In contrast, Cluster Picker is applicable in situations where more closely connected clusters are expected in the studied populations.

Transmission network analyses are critical for making inferences about HIV-1 spread and patterns of mixing between different populations, important for the design of effective interventions.^1–4^ Several tools have been developed to infer HIV-1 transmission networks and majority of these rely on genetic distances (GDs) between nucleotide sequences, patristic distances, phylogenetic subtrees, or simulated data.^5,6^ HIV-TRAnsmission Cluster Engine (HIV-TRACE)^7^ and Cluster Picker^8^ are currently among the most popular and widely used programs in HIV-1 transmission network analysis. In this study, we expound on a previous study^9^ that evaluated the performance of these tools by looking at difference risk groups and analyzing the structure of HIV-1 transmission networks identified by both programs. A total of 2,017 HIV-1 *pol* sequences from cross-sectional surveys conducted in Ugandan fisherfolk communities (FFCs) (*n* = 728) of Lake Victoria, female sex workers (FSWs) (*n* = 592), and general population (GP) (*n* = 697) groups were analyzed for a 7-year period (2009–2016). These populations were surveyed under the Medical Research Council (MRC)/Uganda Virus Research Institute (UVRI) and London School of Hygiene and Tropical Medicine (LSHTM) Uganda Research Unit's Molecular Epidemiology Study that aimed to determine HIV-1 transmission networks in high-risk and GP groups in Uganda. The term fisherfolk was used to refer to persons living in villages that are located along the shores of Lake Victoria, whereas the GP refers to neighboring inland or mainland communities that are mostly agrarian or involved in trade. The term FSWs includes persons involved in either commercial or transactional sex. In this study, transmission networks referred to genetically closely related HIV-1 sequences based on a predefined GD threshold and/or a monophyletic group on a phylogenetic tree with high bootstrap support (>0.95) where two highly similar sequences are known as pairs and more than two sequences as clusters.^1,4^ In identifying networks, HIV-TRACE calculates the pairwise GDs between HIV-1 sequences, whereas Cluster Picker relies on maximum GD but also considers a high bootstrap support at the common node for closely related sequences on a phylogenetic tree. A GD of 1.5% was chosen as the optimum threshold for use in this study based on previous evaluations done across different GD thresholds in similar populations.^1,4^ In our previous study, we analyzed HIV-1 sequences with epidemiological linkage data across a range of GD thresholds (0.5%–5%) and considered a maximum GD of 1.5% as an ideal cutoff to identify viral transmission networks in the studied populations.^1^

At a GD cutoff of 1.5%, 52 HIV-1 transmission pairs and 4 clusters of triplets were identified in Cluster Picker. More transmission pairs (*n* = 91) and clusters (*n* = 11; 9 triplets, 1 cluster of 4 individuals and 1 cluster of 5 individuals) were detected at the same GD threshold in HIV-TRACE. This difference in results was attributed to the different algorithms implemented in each program for network identification. HIV-TRACE uses a single-linkage algorithm,^7^ which means that the networks it identifies can be connected by just a few individuals with sparse links, whereas Cluster Picker searches for more closely connected clusters (Fig. 1). A larger proportion of HIV-1 transmission networks were found in the FFCs (>60% of all pairs and >80% of clusters identified in both programs) (data not shown), a population with the highest HIV-1 incidence in Uganda. Figure 1 shows HIV-1 transmission networks identified in HIV-TRACE and Cluster Picker from the same data sets comprising HIV-1 sequences from different risk groups. In Figure 1C, we delineate the structure of networks identified by both programs to examine the characteristics of networks identified by both tools.

**FIG. 1.**
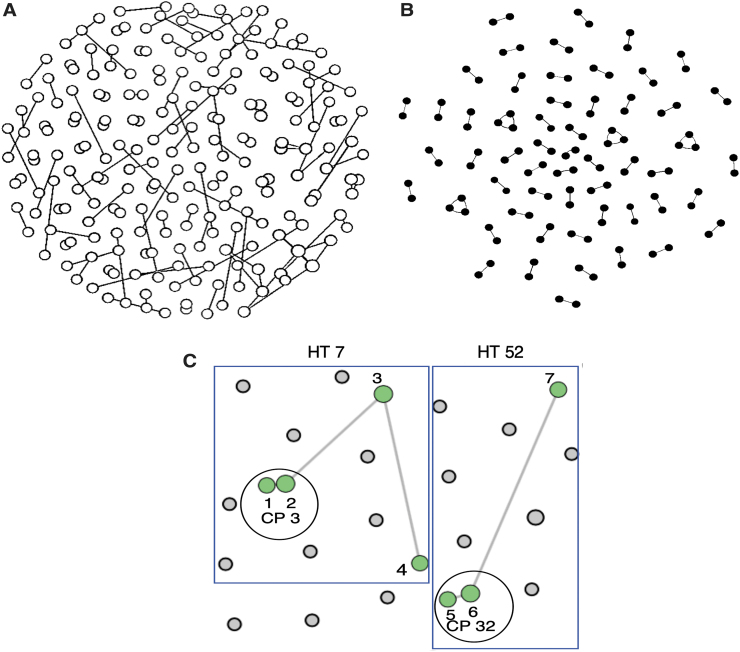
Transmission networks detected by HIV-TRACE and Cluster Picker. The *circles* in each panel represent nodes (each node represents an HIV-1 sequence from an individual) in the network and the edges show the connections between adjacent or sparse nodes. **(A)** Shows a network of HIV-1 sequences in HIV-TRACE. The *white circles* correspond to clustered nodes and the overlapping edges represent connections for distantly linked nodes relative to others in the network. **(B)** Shows a network identified in Cluster Picker from the same data set and the *black circles* correspond to clustered nodes. **(C)** Shows the same networks identified in both HIV-TRACE [HT 7 (nodes 1–4) and HT 52 (nodes 5–7), outlined by the *blue boxes*] and Cluster Picker [CP 3 (nodes 1 and 2) and CP 32 (nodes 5 and 6), outlined by larger *circles* around clusters]. In the two networks, the *green circles* represent linked nodes in a pair or cluster. The *gray lines* represent links between nodes of a cluster, with *longer lines* indicating more sparsely distant connections relative to other nodes in the network. The *gray circles* represent nodes of clustered HIV-1 sequences in the network that have been collapsed. CP, Cluster Picker; HT, HIV-TRACE; HIV-TRACE, HIV-TRAnsmission Cluster Engine.

In both networks, more closely connected nodes (numbered 1, 2, 5, and 6) that comprised transmission pairs were detected by Cluster Picker, whereas additional nodes (numbered 3, 4, and 7) that linked to other external or sparse nodes were detected by HIV-TRACE. This finding suggests that Cluster Picker may be more preferable for use in more homogenous populations such as the FFCs where recent ongoing HIV-1 transmissions are common and where HIV-1 sequences are likely to cluster more densely. In contrast, HIV-TRACE may be more suited for use in populations that are more diverse and where the sampling could span for a longer period of viral transmission. Nonetheless, in both programs, a greater proportion of HIV-1 transmission networks consisted of pairs with fewer larger clusters, which would be expected in a mature and generalized HIV epidemic similar to that in Uganda.

We identified more and larger clusters with HIV-TRACE than Cluster Picker at a maximum GD of 1.5%. HIV-TRACE and Cluster Picker both exploit the GD between HIV-1 sequences to detect transmission networks although Cluster Picker additionally uses branch support values on phylogenetic trees to confirm linkages. However, bootstrap values can change as additional sequences are added to a tree, which could cause clusters to diminish when revisiting a data set upon including new sequences. This feature is absent in HIV-TRACE, which makes it a more suitable program for tracking clusters over time. Also, because HIV-TRACE uses a single linkage approach and rapidly computes pairwise GDs between individual sequences, it does not have to store large matrices and can quickly process very large data sets.^2,7^ As both programs use different algorithms to identify linked sequences, HIV-TRACE detected more and larger clusters compared with Cluster Picker at a GD of 1.5%. However, in a separate study^9^ that evaluated the performance of both programs across a range of GD thresholds (1%–5.3%) using a mixed data set that included next-generation sequence data, HIV-TRACE tended to detect fewer clusters than Cluster Picker at lower GD thresholds (<3%). In this study, the performance of both programs was evaluated at a single GD cutoff determined as ideal for use in our study setting.^1,4^

A limitation of this study is that the comparison between HIV-TRACE and Cluster Picker was performed on sequence data where the clusters were not already known. Additional comparison using data with known clusters would further inform the network analysis and is a consideration for our future analyses. It is also important to note that the results presented in this study and those from a previous investigation^9^ were based on Ugandan HIV-1 sequences that may not be representative of other populations elsewhere in the world.

In conclusion, HIV-TRACE was generally found to detect more and larger clusters than Cluster Picker at a GD of 1.5%. Furthermore, HIV-TRACE detected clusters that were connected by sparse links in the transmission chains. This implies that the choice of the GD threshold applied should put into consideration the characteristics of the population being investigated.^1,2^ Therefore, choosing the right program to use for network identification should depend on several factors that include the aims of the study, the dynamics of the disease epidemic in a particular population, and the type of sampling design.^10^

## Sequence Data

Study nucleotide sequences have been deposited in GenBank with accession numbers MG435358–MG436769. Accession numbers for other sequences used, MG434786–MG435347, JX498971–JX498972, JX498976–JX498990, and JX498992–JX499018.
